# Lateral Habenula Inactivation Alters Willingness to Exert Physical Effort Using a Maze Task in Rats

**DOI:** 10.3389/fnbeh.2021.652793

**Published:** 2021-08-10

**Authors:** Joshua P. Sevigny, Emily N. Bryant, Érica Encarnacion, Dylan F. Smith, Rudith Acosta, Phillip M. Baker

**Affiliations:** Department of Psychology, Seattle Pacific University, Seattle, WA, United States

**Keywords:** discounting, depression, effort, decision-making, reward

## Abstract

An impairment in willingness to exert physical effort in daily activities is a noted aspect of several psychiatric conditions. Previous studies have supported an important role for the lateral habenula (LHb) in dynamic decision-making, including decisions associated with discounting costly high value rewards. It is unknown whether a willingness to exert physical effort to obtain higher rewards is also mediated by the LHb. It also remains unclear whether the LHb is critical to monitoring the task contingencies generally as they change, or whether it also mediates choices in otherwise static reward environments. The present study indicates that the LHb might have an integrative role in effort-based decision-making even when no alterations in choice contingencies occur. Specifically, pharmacological inactivation of the LHb showed differences in motivational behavior by reducing choices for the high effort (30cm barrier) high reward (2 pellets) choice versus the low effort (0 cm) low reward (1 pellet) choice. In sessions where the barrier was removed, rats demonstrated a similar preference for the high reward arm under both control and LHb inactivation. Further, no differences were observed when accounting for sex as a biological variable. These results support that effort to receive a high-value reward is considered on a trial-by-trial basis and the LHb is part of the circuit responsible for integrating this information during decision-making. Therefore, it is likely that previously observed changes in the LHb may be a key contributor to changes in a willingness to exert effort in psychiatric conditions.

## Introduction

Changes in motivation and willingness to exert physical effort are noted across psychiatric conditions including Major Depressive Disorder, addiction, and chronic fatigue (Cohen et al., 2001; Sharpe and Wilks, 2002; Demyttenaere et al., 2005; Bachleda and Darhiri, 2018). For example, patients with Major Depressive Disorder report trying harder even though they objectively exert less effort (Cléry-Melin et al., 2011). Understanding the neurobiological causes of changes in willingness to exert effort to obtain rewards can offer insight into addressing this debilitating aspect of psychiatric conditions. Prior research has revealed a role for the anterior cingulate cortex, the amygdala, the nucleus accumbens, and the dopamine and adrenergic systems in tasks that assess physical effort in rodents (Salamone et al., 1994; Floresco and Ghods-Sharifi, 2007; Bardgett et al., 2009; Yohn et al., 2015).

Common to many of these brain areas is a functional connection with the lateral habenula (LHb) (Lecourtier and Kelly, 2007; Quina et al., 2015). The lateral habenula is proposed to be a key integrator of ongoing context into a wide range of decision behaviors due to its unique position between frontal and midbrain regions involved in motivation and motor behavior (Sutherland, 1982; Lecourtier and Kelly, 2007; Baker et al., 2015). The LHb has also been connected to a growing list of psychiatric conditions (Lecca et al., 2014; Zhang et al., 2017; Schafer et al., 2018). Further, manipulation of the LHb has relieved some of these conditions in human pilot studies (Sartorius et al., 2010; Zhang et al., 2019; Wang et al., 2020), although further research is required.

To date, it remains unclear whether the LHb is involved in the willingness to exert effort during dynamic decision-making tasks. The LHb is known to play a central role in aversive and reward-oriented behavior, particularly when outcomes change dynamically (Baker and Mizumori, 2017; Flanigan et al., 2017; Lecca et al., 2020). The majority of previous work has demonstrated that manipulation of the LHb results in disrupted behavior when outcomes are changed dynamically but not when task outcomes remain stable (Stopper and Floresco, 2014; Baker and Mizumori, 2017). For example, in an operant chamber version of delay or probability discounting, rather than rats altering behavior-based changes in probability for a high reward or a delay to high reward, LHb inactivation resulted in chance performance (Stopper and Floresco, 2014). However, when the discrimination between the high and low reward was tested without changing contingencies, the same rats performed similarly to controls.

The present study sought to examine whether the LHb is involved in a trial by trial consideration of a willingness to exert effort, or more generally, in the ability to discriminate rewards specifically when contingencies change in an unpredictable manner. To test this, we used a unique behavioral paradigm that requires rats to climb a physical barrier in order to receive a large reinforcement or to opt for a smaller reward without the need to climb a barrier. Importantly, the location of the high reward, high effort arm remains constant throughout the task. This allowed us to examine the willingness to exert effort straightforwardly in an ethologically relevant manner. If the habenula is required to integrate a willingness to expend effort to obtain a high reward on a trial-by-trial basis, we should observe changes in behavior when the LHb is inactivated. Alternatively, if the habenula is only required to recognize an alteration in contingencies, then no changes in behavior should be observed. Either result would serve to clarify the larger role the LHb plays in behavioral selection under conditions of physical effort and reinforcement more generally.

## Materials and Methods

### Animals

The rats acquired for this study were 12 female and 12 male *Sprague Dawley* rats from Envigo Labs. All experimental protocols were approved by the Institutional Animal Care and Use Committee at Seattle Pacific University (protocol # 201819-05-R). After a minimum of five days from entry into the lab, rats were handled daily and food-restricted to approximately 85% of their free-feeding weight. Once rats’ weights were stabilized, they began discrimination training on a plexiglass maze.

### Apparatus and Training

**FIGURE 1:**
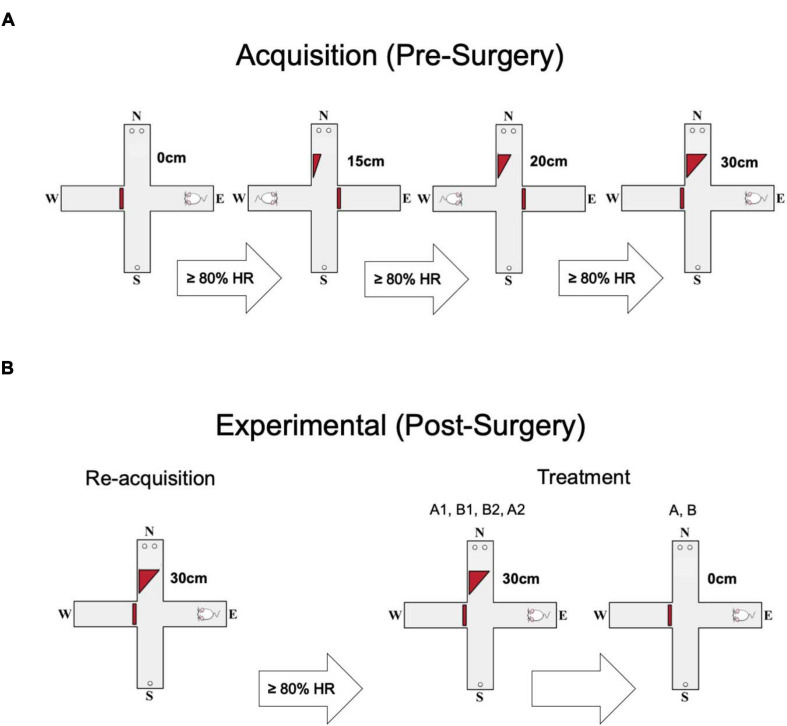
Behavioral protocol. **(A)** Each animal went through four stages of acquisition, progressing to the next ramp height (0 cm, 15 cm, 20 cm, 30 cm) after making ≥80% high reward (HR) choices. The animals’ starting arm varied pseudorandomly. **(B)** Each animal was required to pass a short re-acquisition phase post-surgery, but before experimental trials. Again they were required to make ≥80% HR choices. The experimental phase consisted of two parts. Each animal did two control sessions (A1, A2) and two inactivation sessions (B1, B2). Then, the ramp was removed and the animals did two more sessions, one inactivation and one saline (A, B).

The maze consisted of four arms 5.5 cm in width, 60 cm in length and with walls 15 cm high. The arms were arranged in a plus shape with blocks preventing access to a given arm resulting in a T-shaped maze (Figure 1). Initially, all arms of the maze were baited with 1 sucrose pellet (45 mg pellet, Bio Serve F0042), and rats were placed in a random arm and allowed to explore until consuming all pellets. Once the rats explored and consumed the pellets at least seven times in less than 15 min for two consecutive days, they were advanced to discrimination training. In the discrimination training, the rats were initially trained to discriminate between two choice arms (designated N and S) containing either 1 or 2 sucrose pellets. The location of the high reward was counterbalanced between rats. The remaining arms (E and W) were used as start arms and were pseudo-randomly alternated on each trial with the other blocked off with a plexiglass barrier. Rats were trained daily on 30 trials in this stage until they chose the high reward on at least 80% of trials for two consecutive days. Toward the end of the first week, the rats learned the initial task, reaching two consecutive days with 80% or greater high reward choices. The rats would then progress to the next stage wherein a 15 cm ramp was placed in the arm containing the high reward. The same acquisition criterion was used in this and all following stages as the rat progressed through a further 20 cm and a final 30 cm barrier. The rats had to reach the criteria of >80% for all barriers to be ready for surgery (Figure 1A).

### Surgery

Following completion of training on the 30cm ramp, rats were returned to free feed and then given surgery to place a bilateral cannula in the LHb to inactivate it during subsequent tests using the GABAa agonist muscimol (Sigma). Briefly, rats were placed in an induction chamber and anesthetized with vaporized isoflurane (5%) prior to surgery. Rats were then placed in a stereotaxic apparatus and maintained on isoflurane with a nose cone (1–3%). Once the skull was exposed, four partial pilot holes were drilled and screws were inserted into each. These acted as anchors to keep the headcap and cannula firmly in place. Two holes were drilled bilaterally and the guide cannula was inserted (A–P: -3.5, M–L: ± 0.9, and D–V: 4.35 mm) dorsal to the LHb. Dental acrylic was used to secure the guide cannula to the anchor screws, completing the headcap. Analgesic (Meloxicam) was administered subcutaneously prior to rats waking up and 24 h after surgery.

### Testing and Inactivation Procedure

Once recovered from surgery, subjects went through a short re-acquisition phase where they again had to demonstrate a preference of >80% for the high reward with the 30cm ramp in place (one day). Once completed, rats were moved to the treatment phase of the experiment. Treatments were administered in *ABBA* order. *A* was an injection of vehicle (saline) and *B* was the GABAa receptor agonist muscimol (50 ng per side). Infusions were administered 5 min prior to a test session. A total volume of 0.25 uL was injected at a rate of 0.15 uL/min with a microinfusion pump (74,900 Series Cole Palmer) loaded with two identical 10-uL syringes. The infusion traveled through polyethylene tubing to a 32-gauge injection cannula which extended 1 mm below the guide cannula. The cannula was left in place for an additional minute to allow for full diffusion, after which the cannula was removed. Seven minutes later the subject completed a full session of 30 trials with the 30 cm ramp. Rats were run each day through the sequence until completed. Directly after the ABBA sequence, a second inactivation trial and a second saline trial were conducted with the ramp completely removed. This was done in order to confirm that any observed alteration in high reward preference was due to the presence of the ramp and not due to an inability to discriminate between the reward contingencies. The time to complete each session (session duration) was recorded as an indirect measure of gross motor function.

### Histology

Post-experiment, rats were euthanized by carbon dioxide-induced hypoxia. Following respiratory cessation, rats were perfused first with a 0.9% saline solution and then with a 4% formaldehyde solution. Brains were removed, sliced with a cryostat into 40um sections, and mounted on slides. Slices were stained with cresyl violet (Sigma-Aldrich) in order to visually confirm cannula placement. Any rats that did not have bilaterally accurate placements of internal cannula tips within the borders of the habenula were not included in the analysis.

### Analysis

Statistical analysis was conducted using JASP v 0.14.1 (JASP Team, 2020). Data visualizations were crafted in R v 4.0.2, in R-studio v 1.3.1073, using packages ggplot2 v 3.3.2, and readxl v 1.3.1. (Wickham, 2016; R Core Team, 2017; Wickham et al., 2019). Figures were arranged using Microsoft PowerPoint for Mac v 16.43.

A mixed model, two-way, repeated measures ANOVA was conducted to compare high-reward data across order, treatment and sex. A *post hoc* Student’s *t*-test was used to compare high-reward in combined drug and saline groups. A single sample Student’s *t*-test was used to specifically compare the muscimol, with ramp treatment to a chance outcome (50% high reward). High-reward data in the no ramp condition was compared across muscimol and saline treatments using Student’s *t*-test. Session duration in the no ramp condition was compared between treatments using Wilcoxen’s signed-rank test. Session duration was similarly compared using a mixed model, two-way, repeated measures ANOVA.

## Results

### Acquisition

**FIGURE 2:**
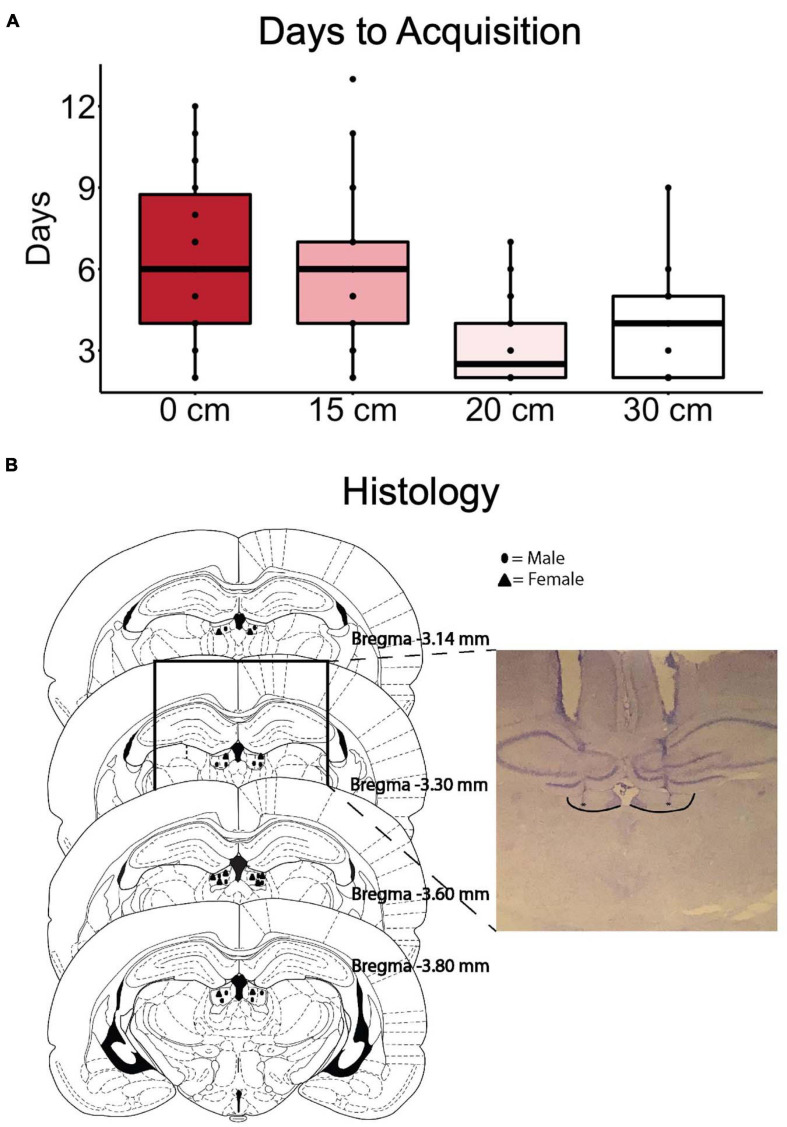
The number of days required to reach criteria (high reward choices ≥80) at each training stage is indicated in **(A)**. **(B)** Summary of correct cannula placements in the LHb with an inset example of a successful placement of cannula aimed at the LHb. Borders of LHb added and (*)’s indicate internal cannula location.

A summary of the initial training performance as the rats progressed through the stages is shown in Figure 2A. Two animals were excluded from the study due to failing to meet the acquisition criterion. After surgery, the acquisition of the 30 cm ramp was repeated as a reorientation, before the treatment sessions (Figure 1B).

### Testing

**FIGURE 3:**
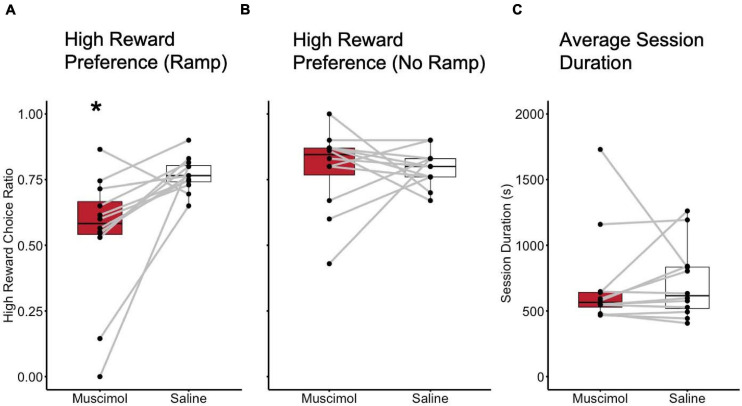
Inactivation of the LHb decreased preference for the high effort, high reward arm. **(A)** High reward choice comparison between inactivation (Muscimol) and control (Saline) sessions with the high 30 cm ramp in place. **(B)** High reward choice comparison between inactivation and control sessions with no ramp in place. **(C)** Session duration compared between inactivation and control sessions. * = *p* < 0.01.

**FIGURE 4:**
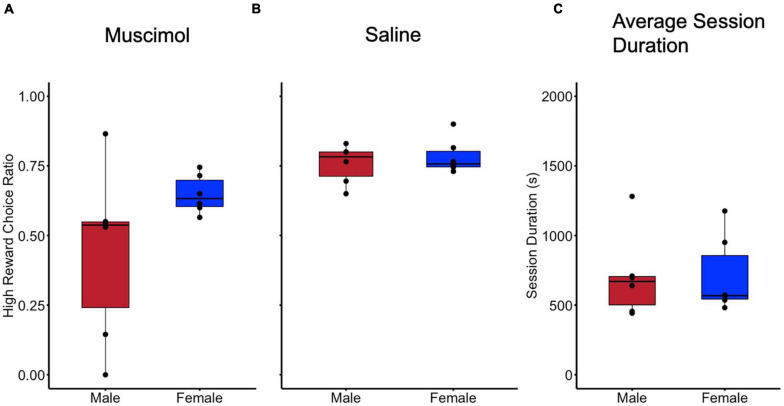
No difference in behavior was observed in reward preferences when considering sex as a biological variable. **(A)** Preference for high reward compared during inactivation (Muscimol) sessions compared by sex. **(B)** Preference for high reward compared for sex during control (Saline) sessions. **(C)** Session duration in seconds for all sessions compared between sex.

Results of the histological examination indicated there were a total of 12 rats with accurate cannula placements (6 male and 6 female) to be included in the analysis (Figure 2B). Because both male and female rats were tested in an ABBA order, a mixed model, two-way, repeated measures ANOVA test of differences was conducted, with treatment and order as repeated measures factors and sex as a between-subjects factor. There were no effects of trial order [*F*(1, 10) = 0.016, *p* = 0.902]. Muscimol inactivation of the LHb significantly decreased choice for the high reward in comparison to saline treatment [*F*(1, 10) = 11.602, *p* < 0.01] (Figure 3A). Due to the significant result in the ANOVA, a *post hoc t*-test, comparing combined drug and saline replicates was performed using the Bonferroni correction [*t*(11) = −3.280, *p* < 0.01, *d* = -0.947] confirming that muscimol treatment significantly reduced high reward, high arm preference. The proportion of high-reward responses did not differ between sex when considering treatment condition, *F*(1, 10) = 2.841, *p* = 0.123 (Figures 4A,B). No interaction effects were observed for order, treatment or sex.

One possibility was that the reduction in choices for the high reward was due to an impaired ability to discriminate reward conditions generally. To control for this possibility, a single sample *t*-test was used to test whether rats differed from chance (μ_o_ = 0.5) in their high-reward choices. Results revealed that high-reward choices did not differ significantly from chance with the muscimol treatment [*t*(11) = 0.624, *p* = 0.545]. High-reward choices did significantly deviate from chance, however, (μ_o_ = 0.5) in the saline condition [*t*(11) = 14.327, *p* < 0.01, *d* = 11.783]. As a follow-up, rats were also tested on the following days without the ramp present to determine whether preferences for the high-reward were altered in the absence of effort as a factor. Student’s *t*-test found no difference in proportion high-reward choice between inactivation and control treatments when no ramp was present in the high-reward arm [*t*(11) = −0.063, *p* = 0.951], indicating that reward discrimination ability remained intact despite the muscimol manipulation (Figure 3B). Wilcoxen’s signed-rank test found no difference in session duration between inactivation and control treatments when no ramp was present in the high reward arm (*W* = 29.000, *p* = 0.456).

To further examine whether LHb inactivation altered other aspects of rats’ performance, especially gross motor function, the time taken to complete sessions (session duration) was examined. A mixed model, two-way, repeated measures ANOVA was used with treatment and order as repeated measures factors, and sex as a between-subjects factor. No difference in session duration was observed in regards to order [*F*(1, 10) = 4.435, *p* = 0.061]. No difference in session duration was observed between inactivation and control treatments [*F*(1, 10) = 0.029, *p* = 0.869] (Figure 3C). There was also no difference in time to session completion observed between sexes, *F(1, 10)* = 0.003, *p* = 0.958 (Figure 4C). No interaction effects with time to complete a session were observed for order, treatment, or sex.

## Discussion

The present study sought to determine whether the LHb is important when animals are required to consider physical effort as a factor in obtaining a higher value reward in an ethologically relevant maze based task. Inactivation of the LHb led to an overall reduction in preference for the high effort, high reward arm. These findings suggest that when animals are faced with an effort-based decision, the LHb is required for optimizing rewards. This was further evidenced as there was no difference between treatments when the ramp was removed, demonstrating that the rats had not forgotten which arm contained the high reward, despite reducing preference to chance levels when the ramp was present. Instead, these results demonstrate a decreased willingness to exert the effort required to obtain the reward even when reward conditions are held constant. Additional analyses revealed there were no differences between the time taken to complete a given session regardless of treatment, indicating that the deficit was likely not due to any motor impairments. In addition, there were no differences in choice preference or magnitude of decrease in high reward choices when accounting for sex as a biological variable.

The use of the maze based effort task reveals an important contribution of the LHb to common behavioral conditions associated with psychiatric conditions. Fatigue, increased perception of effort, and apathy are important behavioral hallmarks in several disorders (Sharpe and Wilks, 2002; Cléry-Melin et al., 2011; Konstantakopoulos et al., 2011). These same disorders also result in alterations of both structure and function of the LHb (Lecca et al., 2014; Schafer et al., 2018; Germann et al., 2020). The present results suggest that novel treatments including deep brain stimulation may lead to a specific alleviation of effort related behavioral symptoms in addition to other noted behavioral aspects of habenular function.

Prior electrophysiological and calcium imaging experiments reveal that the LHb plays an integrative role in the incorporation of context that influences decision-making. For example, the same LHb neurons can encode both action-locking and escape behavior in response to a looming stimulus in mice (Lecca et al., 2020). These signals are likely related to the velocity correlated neurons that have previously been observed while rats demonstrate motivated reward searching (Sharp et al., 2006; Baker et al., 2015). In zebrafish, brain-wide calcium imaging has also revealed that stress recruits neural ensemble activity to drive a transition from active to passive coping (Andalman et al., 2019). This is similar to the observation that increased activity in ventral tegmental area projecting LHb neurons was associated with increased passive coping in response to chronic mild stress (Cerniauskas et al., 2019). Together, these results support the integrative function of the LHb when deciding how to act in response to many contexts including physical effort in the present study. Likely, changes in habenular activity in freely moving animals facilitate changes in choice behavior.

In contrast to many prior studies of behavioral flexibility involving the LHb (Stopper and Floresco, 2014; Baker and Mizumori, 2017), the present study sought to specifically hold task contingencies constant in the task isolating the effort aspect of the task from any need to recognize changes in behavioral requirements. Stopper and Floresco (2014) found that when the requirements of probability or delay were held constant, no changes in behavior related to LHb manipulation were observed. This contrasts with the findings of the present study suggesting that physical effort is specifically considered on a trial-by-trial basis in the LHb as rats perform ethologically relevant tasks such as reward-seeking in a maze based environment.

Decreases in a willingness to exert effort in the maze based effort task are associated with both dopaminergic and adrenergic systems (Bardgett et al., 2009; Mott et al., 2009). Prior work has shown that the LHb has a prominent influence on dopaminergic neural function (Ji and Shepard, 2007). For example, during aversive experiences excitatory drive in the habenula influences the rostromedial tegmental area, which in turn inhibits dopamine neurons. Driving these neurons using optogenetics leads to avoidance behaviors seemingly simulating the aversive experiences (Stamatakis and Stuber, 2012). In addition, norepinephrine modulation in the LHb alters both motor and arousal associated behaviors (Purvis et al., 2018). Considering norepinephrine modulation alters willingness to exert physical effort to obtain reward (Mott et al., 2009), it suggests effort related changes in the LHb could be associated with signaling from norepinephrine.

The present findings suggest a possible common component, namely the LHb, across a wide variety of brain areas important for integrating effort into optimal decision-making. Further, the use of the maze based effort task also clarified that even when task aspects such as level of effort or reward location are held constant, the LHb still contributes to choices on a trial-by-trial basis. In the future it would be interesting to evaluate at what height, beyond the 30cm’s in this study, rats refuse to climb given our high/low reward ratio of 2:1. Inactivation could also be done at different heights to test if size of effect changes. Regardless, present results indicate that effort is indeed an important factor that is integrated with decision-making processes within the LHb. Presently, rats did not adopt a constant strategy of one or the other arm but rather decreased the frequency of choosing the high effort, high reward arm. This is important for comprehending the LHb’s role in using brain states and contextual factors to dynamically make decisions in both health and psychiatric conditions.

## Data Availability Statement

The raw data supporting the conclusions of this article will be made available by the authors, without undue reservation.

## Ethics Statement

The animal study was reviewed and approved by the Seattle Pacific University Institutional Animal Care and Use Committee.

## Author Contributions

JS, EB, ÉE, DS, RA, and PB designed, carried out the experiments, and contributed to writing the manuscript. JS analyzed the data and prepared the figures. All authors contributed to the article and approved the submitted version.

## Conflict of Interest

The authors declare that the research was conducted in the absence of any commercial or financial relationships that could be construed as a potential conflict of interest.

## Publisher’s Note

All claims expressed in this article are solely those of the authors and do not necessarily represent those of their affiliated organizations, or those of the publisher, the editors and the reviewers. Any product that may be evaluated in this article, or claim that may be made by its manufacturer, is not guaranteed or endorsed by the publisher.
